# Porphyrin/Chlorin Derivatives as Promising Molecules for Therapy of Colorectal Cancer

**DOI:** 10.3390/molecules26237268

**Published:** 2021-11-30

**Authors:** Fatima Dandash, David Y. Leger, Mona Diab-Assaf, Vincent Sol, Bertrand Liagre

**Affiliations:** 1 Doctoral School of Sciences and Technology, Lebanese University, Hadath, Beirut 21219, Lebanon; fatimadandash@hotmail.com (F.D.); mdiabassaf@ul.edu.lb (M.D.-A.); 2 Laboratoire PEIRENE EA 7500, Faculté de Pharmacie et Faculté des Sciences et Techniques, Université de Limoges, 2 Rue du Dr Marcland, CEDEX, 87025 Limoges, France; david.leger@unilim.fr (D.Y.L.); vincent.sol@unilim.fr (V.S.)

**Keywords:** porphyrin, chlorin, photodynamic therapy, colorectal cancer

## Abstract

Colorectal cancer (CRC) is a leading cause of cancer-related death. The demand for new therapeutic approaches has increased attention paid toward therapies with high targeting efficiency, improved selectivity and few side effects. Porphyrins are powerful molecules with exceptional properties and multifunctional uses, and their special affinity to cancer cells makes them the ligands par excellence for anticancer drugs. Porphyrin derivatives are used as the most important photosensitizers (PSs) for photodynamic therapy (PDT), which is a promising approach for anticancer treatment. Nevertheless, the lack of solubility and selectivity of the large majority of these macrocycles led to the development of different photosensitizer complexes. In addition, targeting agents or nanoparticles were used to increase the efficiency of these macrocycles for PDT applications. On the other hand, gold tetrapyrrolic macrocycles alone showed very interesting chemotherapeutic activity without PDT. In this review, we discuss the most important porphyrin derivatives, alone or associated with other drugs, which have been found effective against CRC, as we describe their modifications and developments through substitutions and delivery systems.

## 1. Introduction

Colorectal cancer (CRC) is one of the most commonly diagnosed multistage cancers and one of the leading causes of cancer mortality worldwide [1]. In 2018, CRC was the third most common cancer with 1.8 million cases globally, and the second leading cause of oncological death with 862,000 deaths [2]. The five-year survival rate for patients diagnosed with early-stage CRC is approximately 90%, whereas the survival rate for patients diagnosed with advanced-stage CRC is as low as 13.1% [3]. The first-line treatment usually comprises surgical procedures followed by combinational chemotherapy [4]. Despite improvements in the treatment of CRC, the mortality rate of CRC is still high. Thus, there is an urgent need to develop alternative treatments for CRC. Recently, photodynamic therapy (PDT) using, as photosensitizers (PSs), porphyrin or chlorin derivatives is receiving increasing attention as a CRC treatment [5]. PDT, which is an alternative cancer treatment, appears to be a promising option [6]. The molecular mechanism of PDT involves simultaneous interaction between a PS, a light source with an appropriate wavelength, and molecular oxygen. During PDT, PSs absorb visible light and convert energy into surrounding molecular oxygen, generating a range of highly reactive oxygen species (ROS) [7]. Two types of photoreaction mechanisms are invoked to explain PS action: a light-activated PS in its triplet state can generate free radicals via electron or proton transfer (type I photochemical reactions), or singlet oxygen (^1^O_2_) is produced via energy transfer (type II reactions). Singlet oxygen seems to be the major mediator of photochemical cell damage, yet its mechanism of action is not well understood [8]. Relative to traditional therapies, PDT has several advantages, including non-invasive therapy, non-cytotoxic molecules without light activation, and site-specific light treatment which decreases side effects, thus accelerating the healing process.

Regarding CRC, PDT may be a promising unconventional treatment due to the resistance of CRC to conventional treatments. For instance, the most common conventional treatments for CRC include surgical resection, chemotherapy, or radiation therapy. Surgical resection is standardly used until stage III CRC. Patients with stage IV disease often require chemotherapy and/or radiation therapy combined with surgery to treat the disease. However, unfortunately, half of all metastatic CRC patients are resistant to 5-FU-based chemotherapies, which prevents their overall treatment and recovery [9]. This is most probably because CRC cells can upgrade DNA repair mechanisms, deregulate signaling pathways due to oncogene mutations, and promote drug metabolism [10], thus decreasing chemotherapeutic drug-induced apoptosis. Radiation therapy is usually utilized pre-CRC surgical resection in stages II to IV, but some CRC patients also show resistance to radiation therapy [11]. Moreover, radiation therapy has several negative side effects on patients such as nausea, stool leakage, fatigue, sexual problems, skin irritation, rectal irritation and diarrhea [12]. On the other hand, PDT is minimally invasive, has a low morbidity rate, can retain the function of healthy tissues, has fewer side effects, can avoid systematic toxicity, and most importantly has no drug resistance and allows for repeated treatments [13].

Porphyrins are a family of heteromacrocyclic organic compounds, which contain four pyrrole rings interconnected via methine bridges that occur naturally [14]. Chlorins, for their part, are dihydroporphyrin macrocycles that contain one pyrroline ring and three pyrrole rings. Porphyrins and chlorins have been considered extremely attractive for medical uses due to their beneficial photochemical, photophysical and biological properties. Porphyrins have unique optical properties, such as a complex absorption spectrum due to their electronic structure and a fixed absorption wavelength in the visible region [14]. They are fluorescent and characterized by their dark purple color, while metalloporphyrin compounds have different colors depending on the metal ions. Importantly, porphyrins are chemically stable and can stabilize a variety of metal ions [5]. Chlorin properties are very similar; they showed UV–visible spectra with a Q band (Q1) with a wavelength near 650–700 nm and a more significant absorption coefficient.

Initially, scientists discovered the presence of porphyrin in hemes, chlorophylls, and bacteriochlorophylls. Hemes are found in hemoglobins, myoglobins, cytochromes, catalases, and peroxidases, which are responsible for respiration, drug detoxification, and other vital biological actions in humans, whereas chlorophylls and bacteriochlorophylls are responsible for photosynthesis in plants and bacteria [15]. Nowadays, porphyrins have widespread applications, such as in dye-sensitized solar cells [16], preparation of molecular devices [17] and disease diagnosis and therapy [18].

These fundamental roles of porphyrin in our lives led scientists to investigate and research it further with respect to the greatest threat to human health, cancers. In the 1960s, the first promising use of porphyrin was discovered, localizing tumors, as scientists proved its existence in each tumor via adequate fluorescence techniques [15]. Lipson et al. reported the synthesization of hematoporphyrin derivative (HpD) by treating hematoporphyrin chloride with hydrochloric acid and sulfuric acid. In 1978, HpD and argon dye lasers were used to treat skin and canine lung cancer cells [19]. This technique of using a PS (HpD) and a light source for treating diseases, mainly cancer, was called PDT. HpD was then named Photofrin^®^ after neutralization and purification. Furthermore, in 1980, Dougherty, using Photofrin^®^, obtained a complete cure after treating early-stage central type squamous cell carcinoma with bronchofiberscopic PDT [19], which revealed the therapeutic importance of PDT.

PDT is characterized as a non-invasive binary cancer therapy, with good selectivity to tumor tissues and low side effects [20,21,22], that effectively helps in curing and improving the quality of life of cancer patients. PDT can be used with other treatments such as surgery, radiation therapy, or chemotherapy; it can also be used alone for patients who cannot handle the side effects of conventional treatments [23,24]. PDT depends on light irradiation of a tumor-localized PS, to form excited PSs that interact with oxygen molecules and other molecules in the vicinity of the sensitizer such that highly reactive cytotoxic species are produced (e.g., singlet oxygen species, superoxide anions, hydroxyl radicals), which are quite toxic to cancer cells [5]. Due to the ability of porphyrins to selectively localize and remain in various cancer cells and due to their low-dark toxicities [25], they are now among the most-used PSs in PDT. The accumulation of porphyrin in cancer cells is due to π–π stacking between the π-conjugated structure of porphyrin and the aromatic amino acid residues of blood proteins (LDL, albumin, and transferrin), and to the activated endocytosis of these proteins facilitated by receptors activated in cancer cells [26]. To increase the selectivity of porphyrins, several approaches were undertaken, such as modifying the the structural design of porphyrin, since the accumulation of the porphyrin derivatives significantly depended on the functional porphyrin’s position and the substituent’s steric hindrance [26]; using nanoparticle (NP) drug carriers to ensure PS drug solubility and improved passive uptake, with functionalized active targeting abilities (e.g., overexpressed peptides) that could ensure specific uptake in tumor cells to enhance the overall efficacy of PDT [27]; conjugation of the PS drug to specific ligand or biomolecule moieties (monoclonal antibodies (mAb), proteins (e.g., transferrin), nucleic acids (aptamers), small molecules (folic acid), polymers (hyaluronic acid) and peptides (proteins), etc.) which are complementary to overexpressed cancer cell receptors and therefore via a molecular recognition process enhance PS drug uptake in target tumor cells [13]. In addition, porphyrin’s nature and features play a very important role in the efficiency of PDT treatment [28].

Chemotherapy drugs are another type of drug that porphyrins are used in combination with, mainly to enhance the targeting and selectivity of the bioactive groups, thus obtaining the full antitumor effect of the chemotherapy. Different authors proposed new PS complexes containing porphyrins associated by a chemical bond (covalent, ionic, etc.) with chemotherapeutic agents. Some examples of drugs that can have a synergetic effect in a combined treatment of chemotherapy, photothermal therapy and PDT include porphyrin–platinum conjugates [29,30,31,32], porphyrin-5-fluorouracil conjugates [33], porphyrin–nitrogen mustard conjugates [34,35], porphyrin–gold conjugates [36,37,38,39], etc. To increase the water solubility, cellular uptake and subcellular localization of porphyrin-based drugs, porphyrins can be structurally modified with different types of substituents [40]. Importantly, porphyrins can be loaded into nanocarriers to obtain superior targeting.

In this review, we present different examples of porphyrin derivatives as effective PSs alone or with various drug carriers that can be used for CRC treatment.

## 2. Porphyrin and Chlorin Derivatives for PDT Treatment of Colorectal Cancer

### 2.1. Photofrin^®^

Photofrin^®^ was involved in early clinical trials of PDT, where it belongs to the first generation of porphyrins [41]. Photofrin^®^ demonstrated anticancer activity in several cancers [42,43]. A clinical study carried out by Sun et al. explored the recent curative effects and adverse reactions of Photofrin^®^ photodynamic adjuvant treatment on young patients with advanced CRC by comparing the results of the treatment of the observation group (23 patients with advanced CRC who had accepted semiconductor laser photodynamic adjuvant treatment) with the control group (30 patients who had accepted concurrent radiotherapy and chemotherapy) [44]. The observation group, where patients were given 2 mg/kg of Photofrin^®^ and followed by laser irradiation after 48 h, showed an increased survival time, decreased length of hospital stay, a decrease in the number of patients exhibiting symptoms such as hematochezia, change in bowel habits, intestinal stimulation and incomplete intestinal obstruction, and a decreased adverse reaction rate compared to control group [44]. Thus, the curative effects of PDT treatment were significantly higher than those of the conventional treatments and therefore Photofrin^®^ could be used as an adjuvant treatment that effectively enhanced the management of CRC.

Kulbacka et al. wanted to study the effect of Photofrin^®^ on drug-resistant CRC cells, so they tested Photofrin^®^’s oxidative effects on two CRC cell lines (doxorubicin-sensitive (LoVo) and -resistant (LoVoDX) [45]. First, the authors examined the intracellular localization of Photofrin^®^ in treated cells and found that the fluorescence intensity decreased in LoVoDX cells after 4h, while it did not change significantly in LoVo cells. Second, Kulbacka et al. tested the effect of Photofrin^®^ on the oxidative stress factors and the results confirmed that LoVo cells responded better and more quickly to the treatment, as oxidoreductive mitochondrial activity decreased and superoxide dismutase activity increased directly after PDT. However, it took 3 h for the mitochondrial activity to decrease and superoxide dismutase activity to increase. In addition, the level of lipid peroxidation increased with time in LoVo cells, while in LoVoDX cells it remained consistent [45]. Therefore, Kulbacka et al. assumed that combing Photofrin^®^ with multiple drug resistance modulators would be a good option to obtain better PDT anticancer effects.

In another study investigating resistance to PDT, Liu et al. studied the anticancer effects of Photofrin^®^ in p53 mutated (HT-29, DLD1 and SW480) and p53 wild-type (HCT116, LoVo and RKO) CRC cells [46]. The experiment results concerning cell viability, tumor size and survival percentage showed a greater increase in cell viability inhibition in p53^wt^ CRC cells than in p53^−/−^ HCT116 cells, a higher increase in the tumor volumes in p53^mut^ and p53^−/−^ cells than in p53^wt^ and p53^+/+^ cells, and a better survival percentage in RKO (p53^wt^) and p53^+/+^ HCT116 cells than in HT-29 (p53^mut^) and p53^−/−^ HCT116 cells. In addition, Liu et al. proved that after p53 mutation or deletion, the expression levels of five p53-regulated miRNAs (especially miR-124) was downregulated and the expression of the inhibitor of apoptosis-stimulating protein of p53 (iASPP) was encouraged, as they found that p53 enhanced PDT’s effects on CRC cells by promoting miR-124 expression, which inhibits iASPP expression [46]. All in all, p53 as a transcriptional regulator seems to play a key role in increasing the sensitivity of CRC cells to PDT treatment and the miR–iASPP interaction could be a useful pathway to overcome treatment resistance in p53-mutant or -deleted cells.

### 2.2. Tetraphenylporphyrin and Their Derivatives

**Figure 1:**
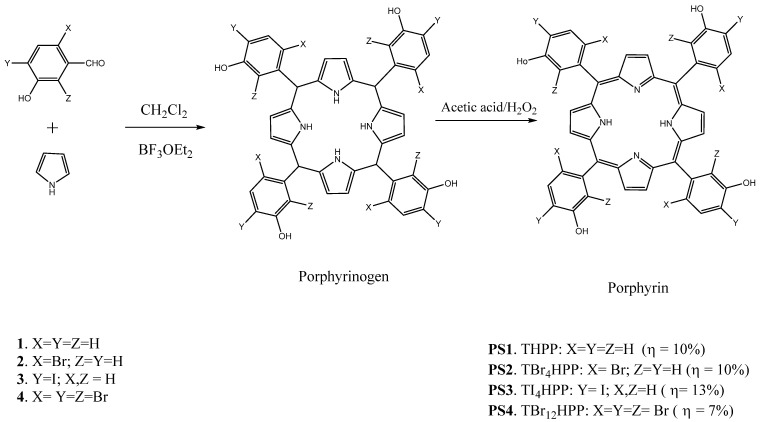
Synthesis of 5, 10, 15, 20-tetrakis(3-hydroxyphenyl)porphyrin derivatives **PS1**–**PS4** [48].

**Figure 2:**
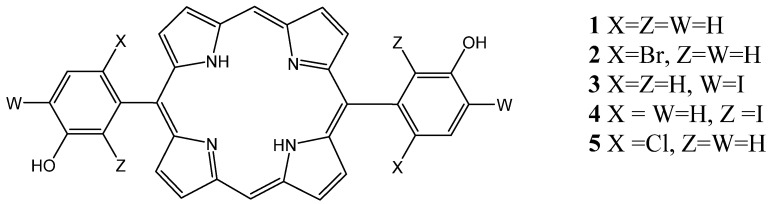
Structures of 5,15-diarylporphyrins used as **PS1**–**5** [49].

One of the synthetic *meso*-substituted porphyrins that has been considered extremely attractive is the lipophilic 5,10,15,20-tetraphenylporphyrin and its derivatives, due to their ease of preparation, large π-conjugation and ability to incorporate a variety of metal ions [47]. In a first study, Serra et al. tested the PDT activity of 5,10,15,20-tetrakis(3-hydroxyphenyl)porphyrin derivatives; the biological activities of the brominated and iodinated derivative sensitizers (**PS1**–**PS4**) (Figure 1) showed that the *para*-substitution by iodine atoms resulted in a poor photodynamic effect due to a decrease in the photostability and hydrophilicity. However, the porphyrin with four bromine atoms in the *ortho*-positions of the phenyl rings (**PS2**) had a high singlet oxygen quantum yield and presented an IC_50_ = 113 nM against the WiDr cell line, which was approximately six times less than the IC_50_ of the Photofrin^®^ [48]. In the second study on 5,15-bis(3-hydroxyphenyl) porphyrin and its halogenated derivatives, Serra et al. showed that all porphyrins resulting from grouping 5,15-diarylporphyrins with different types of halogen substitutions (Figure 2) were more effective than Photofrin^®^ against the WiDr cell line, due to their high ability to generate singlet oxygen, and that the nonhalogenated porphyrin 5,15-bis(3-hydroxyphenyl) showed the best cytotoxicity due to its high cell uptake [49].

**Figure 3:**
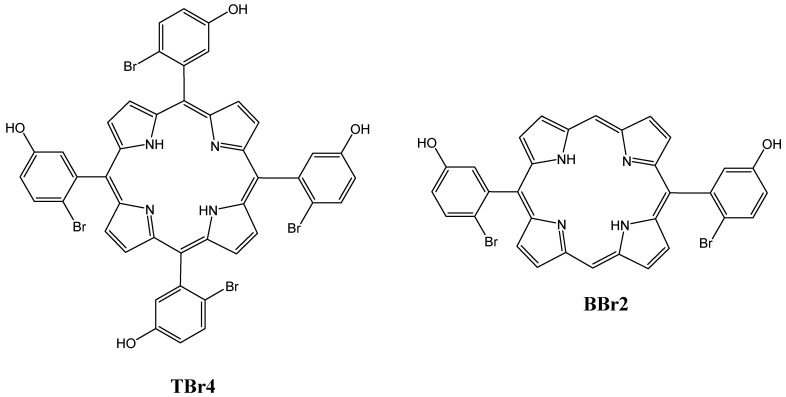
Chemical structures of PS 5,10,15,20-tetrakis(2-bromo-3-hydroxyphenyl)porphyrin (TBr4) and 5,15-bis(2-bromo-3-hydroxyphenyl)porphyrin (BBr2) [50].

The inspiring results obtained by Serra et al. encouraged Laranjo et al. to study the in vitro and ex vivo activities of 5,10,15,20-tetrakis(2-bromo-3-hydroxyphenyl)porphyrin (TBr4) and 5,15-bis(2-bromo-3-hydroxyphenyl)porphyrin (BBr2) (Figure 3) against human colon adenocarcinoma. Being lipophilic compounds, TBr4 and BBr2 were localized in the mitochondria, but BBr2 was more potent in destroying the mitochondria, generating ROS and inducing cell death, mainly through necrosis with IC_50_ = 180 nM, compared to TBr4 which had IC_50_ = 464 nM after 24 h of treatment. The in vivo experiments confirmed that BBr2 was more effective than TBr4. However, both PSs caused a significant diminishing in cell growth in WiDr xenografts [50]. In order to increase the solubility of diphenylporphyrin (DPP), recently, Roy et al. [51] developed a hydrophilic boxlike synthetic receptor ExBox4+, which rendered DPP soluble in water and modulated the phototoxicity of DPP by trapping it in its cavity and releasing it when required.

**Figure 4:**
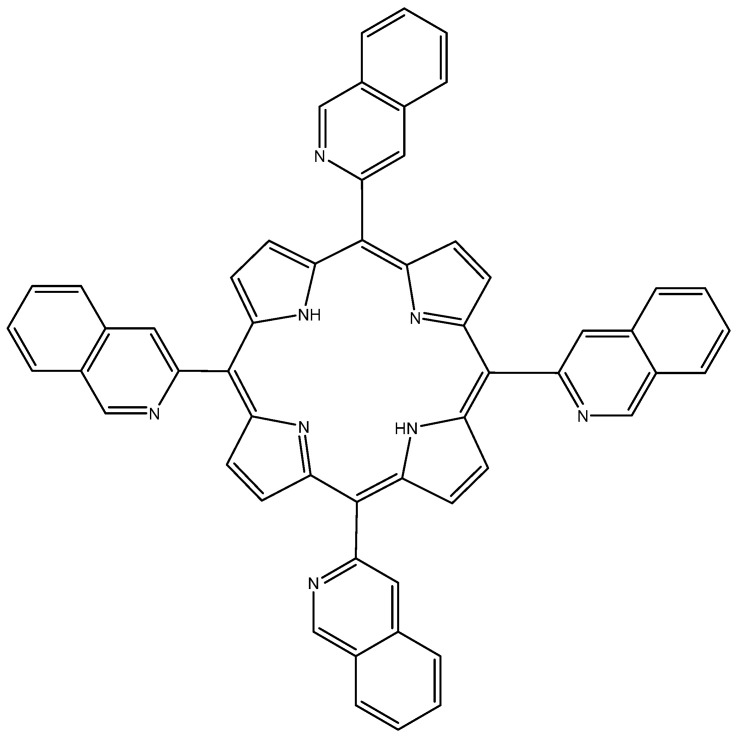
Chemical structure of 5,10,15,20-tetra(quinolin-2-yl)porphyrin (2-TQP) [52].

A more recent study by Costa et al. used the *meso*-substituted porphyrin 5,10,15,20-tetra(quinolin-2-yl)porphyrin (2-TQP) (Figure 4) as a potential PS against HT-29 CRC cells [52]. The added quinoline itself has antitumoral, anti-inflammatory and antimalarial effects [53]. The photophysical characterization of 2-TQP revealed that it had a high quantum yield of singlet oxygen generation (0.62), which was higher than the yield of most PSs used for PDT. Moreover, the photodynamic activity results of 2-TQP against HT-29 cells showed a significant increase in cell death with minimal dark toxicity.

**Figure 5:**
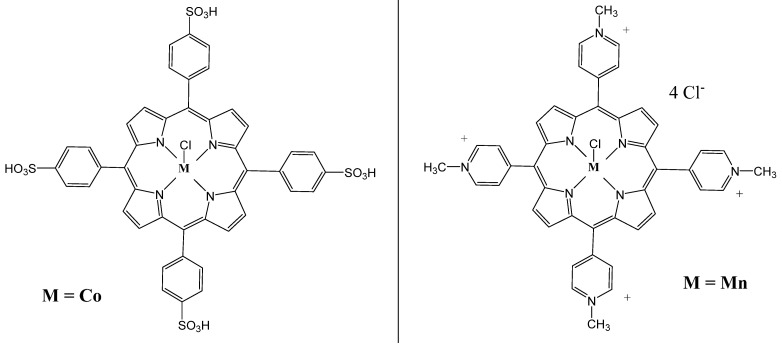
Chemical structures of two metalloporphyrins: CoTPPS and MnTMPyPCl_5_ [54].

In addition, two substituted tetraphenylporphyrins, CoTPPS and MnTMPyPCl_5_ (Figure 5), were tested as anticancer drugs against colon adenocarcinoma, breast adenocarcinoma and human melanoma to explore the power of combining electroporation (EP) and PDT [54]. EP increases drug penetration into cells and thus enhances the efficiency of PDT [50,55]. Experiments were carried out using the two porphyrins, CoTPPS and MnTMPyPCl_5_ against two human CRC cell lines, LoVo and the drug-resistant LoVoDX, which showed that the combined treatment had the best antitumor effects. The results of photodynamic reaction (PDR) experiments showed that treatment of LoVoDX cells with MnTMPyPCl_5_/EP and irradiation with 435 nm resulted in a three-fold decrease in cell viability, as compared to its dark electroporated control. By comparison, the EP–PDR effect using CoTPPS was not significant. Interestingly, the transmission electron microscopy analysis proved that higher cell membrane permeability occurred when electric field intensity increased, without significant changes in the cellular ultrastructure of the cells. These results open an eye on EP–PDR techniques and the importance of the synergistic effect of EP and PDT treatments against multidrug resistance [54].

**Figure 6:**
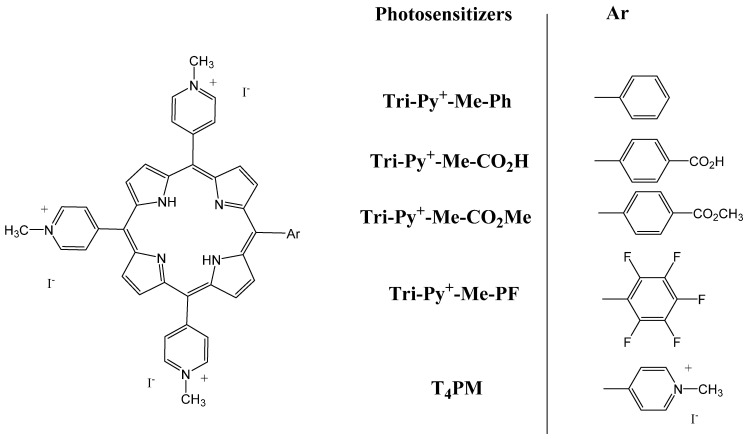
Cationic porphyrin derivatives used for PDT of human colon cancer cells. Chemical structure of 5-phenyl-10,15,20-Tris(1-methylpyridinium-4-yl)porphyrin tri-iodide (Tri-Py^+^–Me–Ph or –Ph); 5-(4-carboxyphenyl)-10,15,20- Tris(1-methylpyridinium-4-yl)porphyrin tri-iodide (Tri-Py^+^–Me–CO_2_H or –CO_2_H); 5-(4-methoxicarbonylphenyl)-10,15,20-Tris(1-methylpyridinium-4-yl)porphyrin tri-iodide (Tri-Py^+^–Me–CO_2_Me or –CO_2_Me); 5-(pentafluorophenyl)-10,15,20-Tris(1-methylpyridinium-4-yl)porphyrin tri-iodide(Tri-Py^+^–Me–PF or –PF); and 5,10,15,20-tetrakis(1-methylpyridinium-4-yl)porphyrin tetra-iodide (T_4_PM) [60].

Positively charged porphyrins are considered extremely attractive for PDT due to their high solubility and ability to penetrate tumor cells [56,57]. In particular, the tetra-cationic compound 5,10,15,20-tetrakis(1-methylpyridinium-4-yl)porphyrin tetra-iodide (T_4_PM) has been widely studied for its therapeutic effects [58,59]. In their study, McCormick et al. aimed to compare the therapeutic efficiency of tri-cationic *meso*-substituted porphyrin derivatives, namely Tri-Py^+^–Me–PF, Tri-Py^+^–Me–Ph, Tri-Py^+^–Me–CO_2_Me and Tri-Py^+^–Me–CO_2_H, with T_4_PM. The five porphyrins (Figure 6) did not show dark cytotoxicity. –Ph, –PF and –CO_2_Me compounds were more photochemically active than T_4_PM and –CO_2_H compounds after light activation, causing cell apoptosis in the human colon adenocarcinoma cell line Caco-2. This study showed that the nature of the peripheral substituent at the *meso*-position is very important for potent photocytotoxicity, and –Ph, –PF and –CO_2_Me porphyrin derivatives were good PSs for PDT [60].

### 2.3. Protoporphyrin IX

5-aminolevulinic acid (5-ALA) is a water-soluble compound, known as a natural precursor of heme. Recently it was found that this compound can also be metabolized to protoporphyrin IX (PpIX), and an increase in ROS production was involved in the cells where PpIX was biosynthetized from 5-ALA [61,62]. Thus, PpIX is one of the important PSs used in PDT and photodynamic diagnosis (PDD) [63]. PpIX has high potential for treating a wide variety of tumors [64,65]. ALA–PDT was tested by Hatakeyama et al. as an anticancer therapy against CRC using LEDs (blue, red and white), which are inexpensive, stable and easier to handle compared to lasers. The results of antiproliferative activity showed that the treated groups had significantly lower cell viability in HT-29 cells, and the blue and white LEDs caused higher cell death than the red LED. The antitumor effect of ALA–PDT using LEDs in a CRC-bearing mouse model was evaluated and the results demonstrated a high tumor inhibition rate (88%) with blue or white LEDs, and a lower rate with red LED. Hatakeyama et al. showed that ALA–PDT can be used to eliminate HT-29 CRC cells in vitro and in vivo [66].

### 2.4. Other Porphyrin and Chlorin Derivatives 

Porphyrin derivatives are highly π-conjugated aromatic molecules with characteristic optical properties, accomplishing an extensive multiplicity of functions in natural and synthetic structures; for example, artificial enzymes in bio-mimetic chemistry, co-factors, and vitamin B_12_ [67].

**Figure 7:**
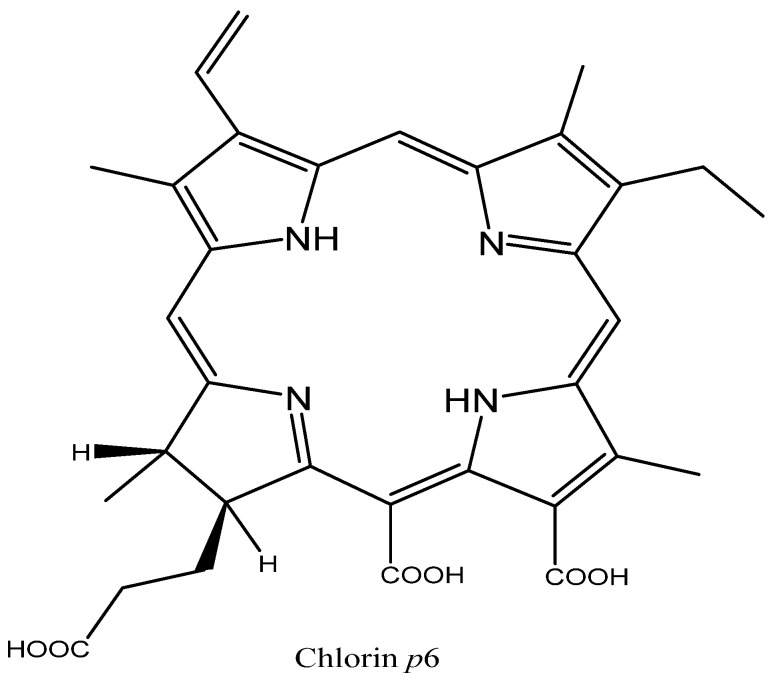
Chemical structure of chlorin *p6* [75].

Chlorins are macrocycles that are given substantial consideration as potential drugs for PDT of cancers [68]. A chlorin, the core chromophore of chlorophyll, is a dihydroporphyrin macrocycle that contains one pyrroline ring and three pyrrole rings [69,70]. Chlorin *p6* has gained considerable interest in recent years [71]. Hence, Sharma et al. studied the effect of extracellular pH on the mode of cell death caused by chlorin *p6* in human colon adenocarcinoma cells (Colo-205) [72,73]. First, the authors found that the percentage of cell death in Colo-205 cells treated with chlorin *p6* (Figure 7) and irradiated in a medium of pH 6.5 was slightly higher than in cells treated in a medium of pH 7.4, most probably due to higher cellular uptake at pH 6.5. However, the fluorescence microscopic images of Colo-205 cells treated with chlorin *p6* when extracellular pH was 7.4 indicated apoptotic cell death, while when extracellular pH was 6.5 the images indicated necrotic cell death. Sharma et al. [72] then found that photodynamic treatment with chlorin *p6* in acidic pH induced more damage to the mitochondrial membrane potential than the same at physiological pH 7.4. In addition, they measured the ratio of ADP/ATP, since mitochondria are the main cellular source of ATP, and found that in cells treated under acidic conditions (6.5) the ratio was significantly higher (5.95 ± 0.35) than the ratio in cells treated under physiological pH (0.41), which correlates with the observed necrosis in the acidic medium. Furthermore, the authors studied the effect of extracellular pH on PDT induced caspase-3 like activity due to the important role of caspase-3 in apoptosis, and found that caspase-3 like activity was higher in cells irradiated at pH 7.4 than at pH 6.5. The authors believe that this increase was because of the inhibition of steps involved in the apoptotic pathway subsequent to caspase-3 activation under acidic conditions, such as inhibition of staurosporine-induced apoptosis under low extracellular pH proved by Terminella et al. [74]. To sum up, chlorin p6 mediated cell death in Colo-205 cells, either by apoptosis when extracellular pH was 7.4 or by necrosis when extracellular pH was 6.5. However, further studies should be conducted to recognize and implement the mechanism of cell death.

Another recent study discussed the mode of cell death induced by two promising chlorin derivatives, *meta*-tetrahydroxyphenylchlorin (*m*-THPC) and verteporfin (VP) [76]. *m*-THPC and VP are two PSs that showed photocytotoxicity against several tumor cells [77,78] and had encouraging results in PDT for human cancers [79,80]. Hence, Song et al. [76] wanted to discover the mechanism by which *m*-THPC-PDT or VP-PDT caused cell death in CRC. The treatment of HCT116 and SW480 cells with *m*-THPC-PDT or VP-PDT led to the inhibition of cell proliferation in a dose-dependent manner, and to an increase in ROS production in a time-dependent manner. The authors proved that *m*-THPC-PDT and VP-PDT treatment of cells could induce autophagy, since it led to accumulation of MAP1LC3B-II, a decrease in the level of SQSTM1/p62, and significant reversal in *m*-THPC-PDT induced autophagy when cells were treated with autophagy inhibitors. Interestingly, Song et al. [76] not only showed that treatment with antioxidants could restraint autophagy and apoptosis, but also that inhibition of autophagy via knockdown of ATG5 or ATG7 could inhibit the apoptosis induced by *m*-THPC-PDT in CRC cells. In vivo experiments showed that *m*-THPC-PDT significantly reduced the tumor volume of HCT116 subcutaneous xenografts, increased the expression of MAP1LC3B-II, and decreased the expression of SQSTM1/p62 in tumor tissues, while these results were reversed when ATG7 was knocked down. Thus, inhibiting autophagy may reduce the antitumor effects of *m*-THPC-PDT. Finally, the authors showed that PDT treatment triggered apoptosis and autophagy by activating the ROS/JNK signaling pathway (*m*-THPC-PDT treatment significantly increased the phosphorylation of JNK, and JNK inhibitor SP600125 markedly inhibited *m*-THPC-PDT- or VP-PDT-induced apoptosis in CRC cells), as it induced autophagy by inhibiting the mTOR signaling pathway (treatment decreased the phosphorylation of p70S6K and mTOR, decreased the levels of SQSTM1/p62 in CRC cells and elevated the autophagic MAP1LC3B-II/MAP1LC3B-I ratio). All in all, *m*-THPC and VP could be potential anticancer drugs that induce apoptosis and autophagy in CRC.

**Figure 8:**
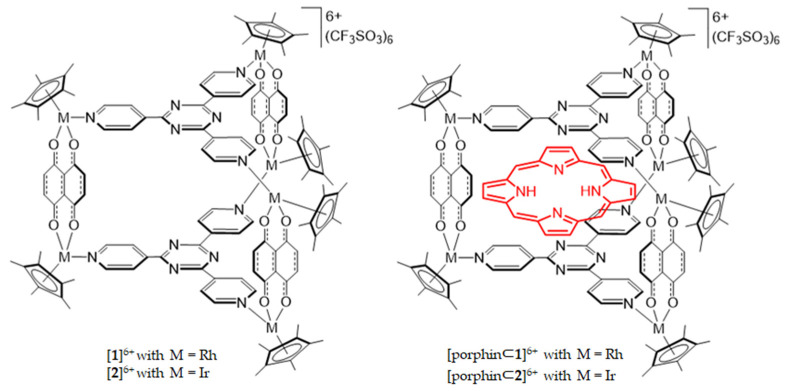
Molecular structures of [**1**]^6+^, [**2**]^6+^, [porphin⊂**1**]^6+^ and [porphin⊂**2**]^6+^ [82].

Garci et al. presented a new strategy using organometallic drug carriers in their study, in which they synthesized three *p*-cymene ruthenium metallaprisms, of general formula (*p*-cymene)_2_Ru_2_(μ4-L)Cl_2_, to transport, shield, and release porphin in HT-29 cells [81]. In vitro results demonstrated that the best arene ruthenium metallaprism among the compounds [2b][CF_3_SO_3_]_6_, [2c][CF_3_SO_3_]_6_, [porphin⊂2b][CF_3_SO_3_]_6_, and [porphin⊂2c][CF_3_SO_3_]_6_ was [porphin⊂2c][CF_3_SO_3_]_6_, since it exhibited great therapeutical performance with LD_50_ = 25 nM and this photoactivity remained optimal for at least 24 h. Two different organometallic cages constructed from Cp*Rh (or Cp*Ir) units of the general formula [(Cp*M)_6_(tpt)_2_(dhnq)_3_] were built by Gupta et al. Porphyrin was encapsulated, resulting in the formation of two compounds [porphin⊂1]^6+^ and [porphin⊂2]^6+^ (Figure 8) [82]. Gupta et al. evaluated the two metallacages as potential delivery systems for PSs against HT-29 cells. The IC_50_ parameter of metallacages, with or without the guest, was approximately 1 μM; however, after irradiation, only the two [porphin⊂cage][ CF_3_SO_3_]_6_ systems showed a PDT effect with IC_50_ less than 5 nM [82]. The two studies revealed that metallacages seemed to be good delivery agents to enhance the effects of PDT against CRC.

### 2.5. Porphyrin/Chlorin Derivatives and Nanoparticles (NPs)

The need for more selective and less toxic delivery systems called the attention of scientists to NPs as carriers for cancer drugs. Targeted delivery via nanomaterials (with diameters ranging from one to 100 nm) of medicines, PTT, PDT, imaging and biosensing for cancer treatment, and medical diagnosis tools has become increasingly important in recent years due to the unique physicochemical properties of NPs [83]. Properties such as excellent surface-to-volume ratio, favorable magnetic and electrical properties, diversity in shape and size, and ideal chemical composition give NPs outstanding potential. In PDT, loads of PSs are lipophilic, insoluble in water, and tend to aggregate, so using NPs as drug carriers can help in solving these problems as they can promote tumor drug concentration and reduce drug side effects [83].

#### 2.5.1. Porphyrin/Chlorin Derivatives and Organic NPs

**Figure 9:**
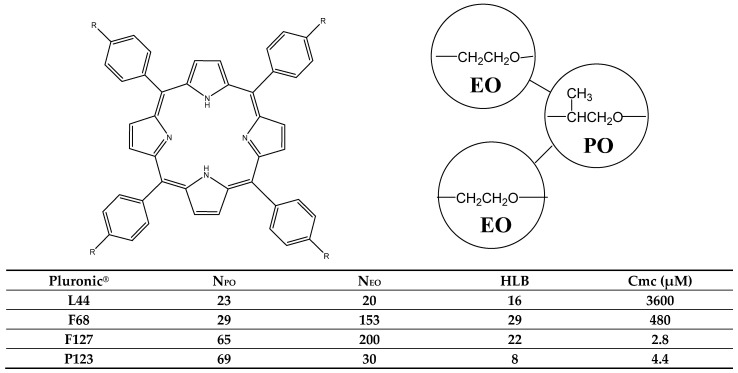
Left side: structural formula of porphyrin PSs possessing different ligands in the *p*-position of the phenyl group: trimethylaminium (TAPP, R = N^+^(CH_3_)_3_); hydroxyl (THPP, R = OH); sulfonic (TSPP, R = SO_3_H); carboxylic (TCPP, R = COOH). Right side: the Pluronic block copolymers L44, F68, F127 and P123. Bottom: average number of ethylene oxide groups (N_EO_), average number of propylene oxide groups (N_PO_), hydrophilic–lipophilic balance (HLB) values and critical micelle concentration (Cmc) values [85].

With the aim of enhancing PDT, two studies explained the construction of two delivery systems. In the first study, Pramual et al. created the first biologically derived, biodegradable polyhydroxyalkanoates (PHA) polymeric NP for delivery of hydrophobic 5,10,15,20-tetrakis(4-hydroxyphenyl)-21*H*, 23H-porphine (pTHPP) [84]. Two different *p*THPP-loaded PHA NPs were constructed via a modified emulsification-solvent diffusion method with different percentages of drug loading. HT-29 cells were incubated with the nanodrug and efficiency tests were performed after 1 to 24 h of incubation and compared to THPP administered in a DMSO solution. Pramual et al. [84] found that the decrease in viability of HT-29 cells incubated with pTHPP-loaded PHA NP increased with time until it reached 93% after 24 h, while the decrease in cell viability of HT-29 cells incubated with PS in DMSO was much faster (90% cell viability decrease after 6 h incubation). The authors assumed that the PHA NPs were safer than the toxic solvent DMSO, and could be a promising delivery system, especially for PDT application [84]. In the second study, Sobczyński et al. aimed to discover the effect of four chemically different types of Pluronics (F127, P123, L44 and F68), as nanovehicles, on the dark cytotoxicity, photocytotoxicity and localization of four model PSs, tetraphenyl porphyrins with the phenyls *para*-substituted by hydroxyl (THPP), carboxyl (TCPP), sulfonate (TSPP) and trimethylamine (TAPP) groups (Figure 9) [85]. Cell viability tests on WiDr cells revealed that cell death increased with increasing Pluronic concentration only for P123 above 20 μM and for L44 above 200 μM, and only TCPP solubilized by F127 above 100 μM had an effect on cell viability. Photocytotoxicity tests of the PSs in the presence of Pluronics after 18 h of incubation of WiDr cells revealed that only Pluronic P123 increased the efficacy of the PSs TAPP and TSPP. However, Pluronics showed encouraging results with respect to intracellular localization and accumulation of PSs [85]. Therefore, Pluronics may act as good solubilizers of porphyrin-based PSs.

In a study aimed at killing CRC cells, Wu et al. used FosPeg^®^, PEGylated liposome vesicles loaded with *meta*-tetra(hydroxyphenyl)chlorin (mTHPC), named Foscan^®^. Chlorin has the same chemical structure as porphyrin but with one less double bond, so the UV–visible spectrum showed a higher optical density in the absorption band with the longest wavelength. This last property rendered it very interesting to use a red light. FosPeg^®^ displayed important in vitro results with HT-29 cells. The lethal doses LD_50_ and LD_70_ of FosPeg^®^ obtained through antiproliferative activity tests were 0.3 and 0.6 μg/mL, respectively, which implies that FosPeg^®^-PDT induced dose-dependent cell death. The Annexin V apoptosis assay at 24 h post-FosPeg^®^-PDT treatment showed that cells underwent apoptosis at lethal doses due to caspase-3 activation. Flow cytometric analysis displayed a time-dependent increase in the sub-G1 portion of HT-29 cells [86]. Taken together, these findings reveal that FosPeg^®^ seemed an effective tool for eradicating CRC cells.

Another drug using targeted PDT was synthesized by Tran et al. The authors synthesized amphiphilic heparin-retinoic acid (HR) and heparin-folate-retinoic acid (HFR) bioconjugates. Both bioconjugates could produce NPs with efficient encapsulation of the PS pheophorbide a (Pha) [87]. However, the HFR bioconjugate had higher Pha loading content and higher efficiency than the HR bioconjugate. The study of the cellular uptake of Pha-loaded NPs via flow cytometry showed no targeting on the folate receptor-negative HT-29 cell line and a targeted anticancer effect on folate receptor-positive HeLa cells. In addition, it was shown that the HFR bioconjugate had a higher anticancer effect on HT-29 cells than the free Pha only. As a result, we can assume that the HFR bioconjugate could be a promising nanocarrier for PDT and chemotherapy.

The second example of combined therapy (photothermal-, photodynamic- and chemotherapy) is the sub-100 nm, SN-38-encapsulated photonic micelles constructed by Yang et al. [88]. SN-38 is an insoluble chemotherapy drug that has good anticancer effects against CRC and other cancers [89,90], but it is difficult to encapsulate in nano-carriers because of its flat aromatic structure. Yang et al. [88] overcame this problem by using photonic nanoporphyrin micelles (NPM), exploiting the intermolecular π–π interactions between SN-38 and the porphyrin. The in vitro tests showed that the SN–NPM drug was stable, had controllable drug-releasing behaviors under laser illumination and high cellular uptake by HT-29 cells, and produced ROS which caused cell death. Importantly, the antiproliferative activity test showed that combination therapy in the HT-29 cell line via SN–NPM increased the in vitro anticancer activity by 78 and 350 times over SN-38 and phototherapy treatments, respectively. The in vivo experiments on mice bearing HT-29 tumor cells confirmed the in vitro results concerning safety, ROS production, photothermal effect and the antitumor effect of SN–NPM, since the SN-NPM+90 J/cm^2^ light group achieved 84.86% tumor growth inhibition [88]. All these findings proved that SN–NPM was a very important anticancer drug with synergistic effects.

**Figure 10:**
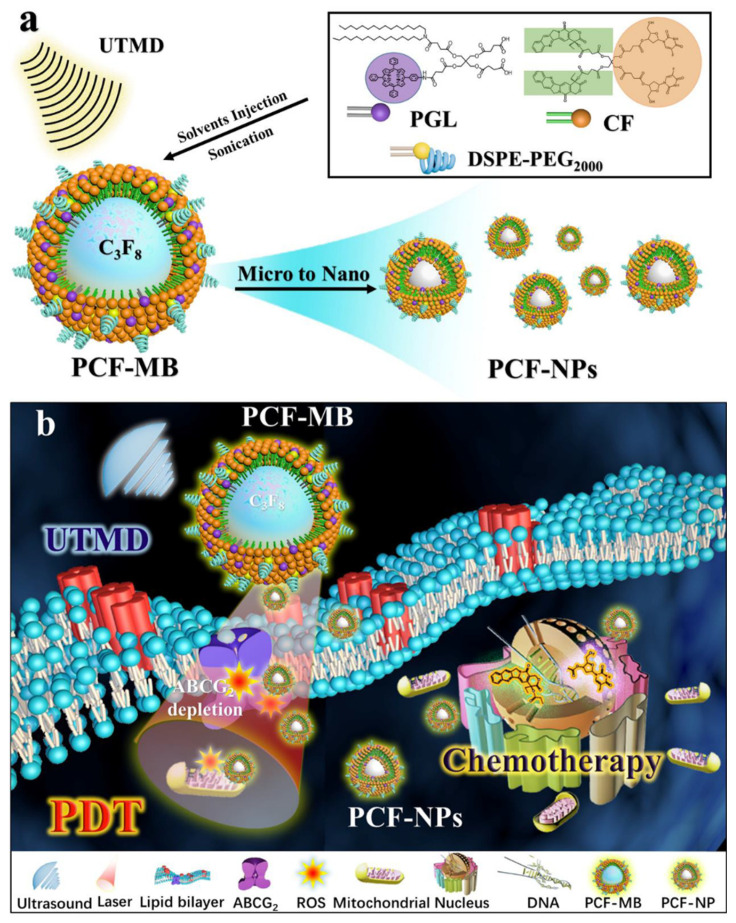
(**a**) Schematic illustration of self-assembly of PCF-MB with porphyrin grafted lipid of PGL and camptothecin-floxuridine conjugate of CF, which can be converted into PCF-NP by ultrasound targeted microbubble destruction. (**b**) PCF-MB mediated chemo-photodynamic combination therapy using the UTMD technique. The therapeutic efficacy may be enhanced, since PDT can deplete ABCG2, by which chemo-drugs can be pumped out of cancer cells [91].

An interesting study regarding multidrug resistance in CRC was conducted by Chen et al. [91]. The authors used Ultrasound (US) to convert a mixture of porphyrin grafted lipid (PGL)/camptothecin–floxuridine (CF) triad microbubbles (MB) into NP. The mixture (PGL-CF) was sonicated to generate PCF-MB via cavitation from perfluoropropane (C_3_F_8_) in the mixture (Figure 10). Local US exposure converted the PCF-MB into PCF-NP under the guidance of US imaging. The aim was to combine chemotherapy promoted by CF with PDT promoted by the PGL to conquer CRC multidrug resistance. In vitro experiments revealed that the drug caused a reduction in the expression of drug efflux transporter ABCG2, which was responsible for cancer drug resistance and an increase in caspase-3 related cell apoptosis. For in vivo results, the PCF-MB + US + light treated group of the HT-29 CRC bearing nude mice showed the most effective results compared to the control groups. In terms of tumor volume growth the drug caused a 90% tumor inhibition rate in the PCF-MB + US + light treated group and the analysis of resected tumor tissues via immunohistochemistry and immunofluorescence showed a remarkable decrease in microvessel count and ABCG2 expression, increase in tumor inhibition rate, and normalizing vascularisation [91]. According to these results, the assembled PCF-MBs might be considered a multi-modal therapeutic agent for synergetic Chemo-PDT that can work through CRC drug resistance.

**Figure 11:**
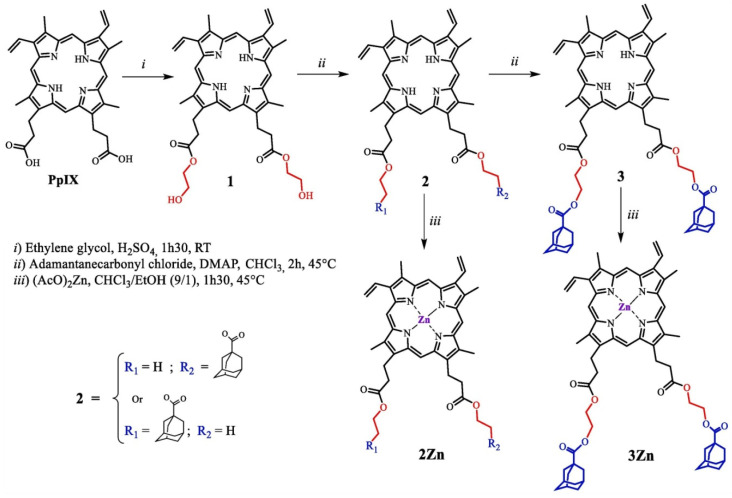
Synthesis of adamantane–PpIX derivatives **2Zn** and **3Zn** [92].

A recent work performed by our laboratory discussed the design and synthesis of two zinc PpIX adamantane derivatives (**2Zn** and **3Zn**) (Figure 11) that are loaded into cellulose nanocrystals (CNCs) to enhance their water-solubility and their cellular bioavailability and can be used as anticancer PDT drugs [92].

**Figure 12:**
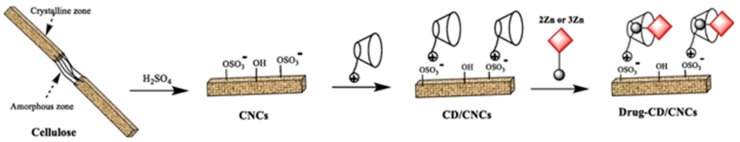
General strategy of CNCs synthesis and encapsulation of **2Zn** and **3Zn** by CD/CNCs [92].

CNCs were obtained from cellulose microfibrils (hydrophilic cotton) after sulfuric acid hydrolysis. After dialysis, their chemical structure showed the presence of some anionic groups (sulfate group) at the C-6 position of some glucose units. Therefore, they could bind cationic cyclodextrin (CD) by ionic interaction. Subsequently, due to the affinity of the hydrophobic cavity of CD for nonpolar molecules, the CD–CNC ionic complexes could be loaded with mono- or di–adamantane–zinc–PpIX(**2Zn**–CD/CNC and **3Zn**–CD/CNC) (Figure 12). In vitro experiments were performed to test the efficiency of the synthesized complexes and the importance of vectorization via CNC. In vitro phototoxicity of **2Zn** and **3Zn** compounds, as well as **2Zn**–CD/CNC and **3Zn**–CD/CNC complexes, was evaluated against the HT-29 cell line using the MTT method with the concentrations of PS ranging from 0.25 to 2.5 μM during 48 h. In the dark, there was no significant cytotoxicity for all PSs, while under illumination the cytotoxicity of PSs was much stronger, especially the encapsulated ones, such that **3Zn**–CD/CNC was the most active complex with IC_50_ = 0.42 ± 0.02 μM. In addition, a flow cytometry analysis was performed to study the effect of the vectorization. The fluorescence of the Zn-PpIX derivatives analyzed via flow cytometry coupled with the AMNIS^®^ image analysis showed that the **2Zn**–CD/CNC and **3Zn**–CD/CNC complexes were much more internalized than **2Zn** and **3Zn**. Therefore, Ndong Ntoutoume et al.’s success in the synthesis of zinc PpIX-adamantane/CD/CNC complexes with good anticancer activity against HT-29 cancer cell line awakens the possibility of using these complexes (CD/CNC) as nanocarriers in various diseases [92].

**Figure 13:**
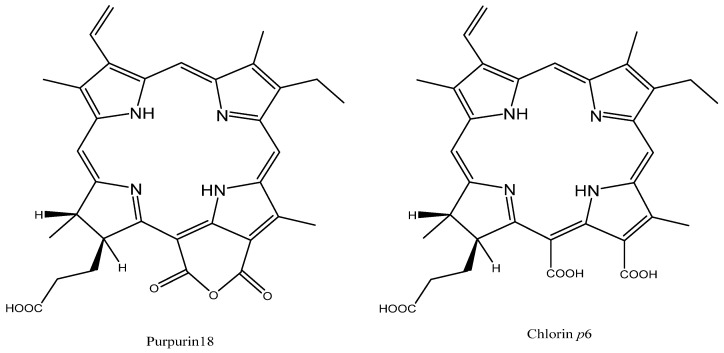
Chemical structure of purpurin **18** and chlorin *p6*.

In another study, Sharma et al. explored the photodynamic potency of a porphyrin derivative, purpurin-**18** (Pp18), when incorporated into phosphatidylcholine liposome against Colo-205 cells [93]. Pp18 was incorporated into liposomes at pH 6.0–6.5 to prevent its conversion to chlorin *p6* inside the cells (due to the presence of an anhydride ring in Pp18) (Figure 13) and to have better cellular uptake. The in vitro studies showed that treatment with 6 µM Pp18 liposome preparation caused 60% higher cell death, with significant damage to both mitochondria and lysosomes, than when Colo-205 cells were treated with 10 µM chlorin *p6*. In addition, changes in cell morphology and DNA fragmentation revealed that the mode of cell death induced by Pp18 liposome depended on irradiation time (longer irradiation time caused necrosis). Importantly, the authors demonstrated that the phototoxic effect of 700 nm radiation was higher at low pH (which favors the presence of Pp18) than at physiological pH. Taken together, these results indicate that Sharma et al. succeded in stabilizing Pp18 by using low pH liposomes, and since tumor tissue pH is known to be slightly acidic, we can benefit from the pH-dependent conversion of Pp18 to chlorin *p6* in order to increase the effects of PDT in colon tumors, but in vivo studies should be performed to confirm the results [93].

#### 2.5.2. Porphyrin/Chlorin Derivatives and Inorganics Nps

**Figure 14:**
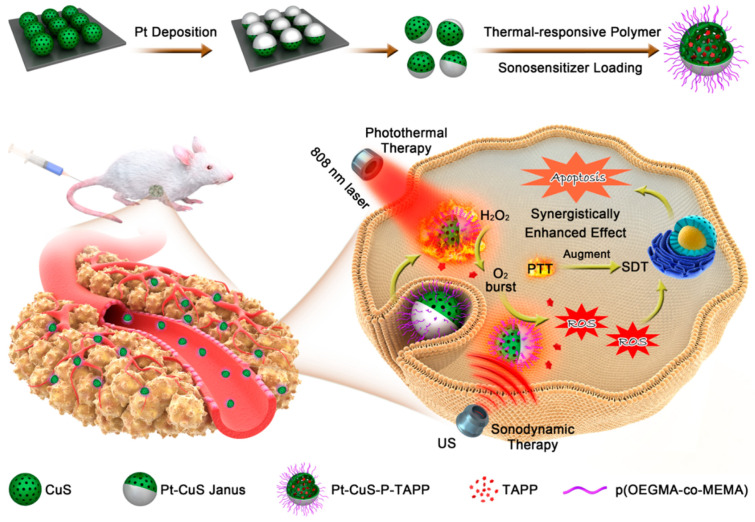
Schematic illustration of the main synthesis procedures and antitumor mechanism of PCPT [95].

Inorganic NP drug delivery systems attracted considerable research interest in the field of PDT due to their great biocompatibility, easy synthesis, stability and remarkable physical-chemical properties involved in boosting PDT therapeutical effects [94]. Liang et al. synthesized a nanomaterial against CRC, labeled PCPT NPs, where the porphyrin complex (tetra-(4-aminophenyl) porphyrin, TAPP) was loaded into hollow structural Pt-CuS Janus NPs composed of hollow semiconductor copper sulfide (CuS) and noble metallic platinum (Pt) (Figure 14) [95]. Interestingly, Pt-CuS Janus played a very important role in increasing the therapeutical efficiency of TAPP, and when combined with ultrasound (US)-triggered sonodynamic therapy (SDT), which has a higher tissue-penetrating depth, was more effective than PDT with photothermal therapy (PTT) promoted by Pt. The in vitro results of the PCPT combined with US and 808 nm laser irradiation group were quite impressive, where cell viability of CT26 murine CRC cells assessed with 2,7 dichlorofluorescein diacetate (DCFH-DA) decreased to 19.8%, and cell apoptosis assessed by live and dead cell staining assays and flow cytometry was significantly induced as a result of beneficial ROS generation. Furthermore, the in vivo results illustrated that the nanodrug was safe and showed, utilizing HIF-1α and VEGF immunohistostaining assay, that mouse tumor slices extracted from the PCPT + US + laser group displayed a very weak fluorescence compared to the control groups, a clear indication of reduced hypoxia and prevention of the increase in VEGF in the tumor; hence the PCPT + US + laser treatment could lessen hypoxia-associated resistance in CRC therapy. Moreover, using the CT26 xenograft tumor model, it was demonstrated that the nanodrug could inhibit tumor growth, since the tumors in the PCPT + US + laser group were completely eradicated without reoccurrence with no significant differences in body weight between the control and the treatment groups. All these results suggest that PCPT NPs may be a safe potential treatment against CRC.

**Figure 15:**
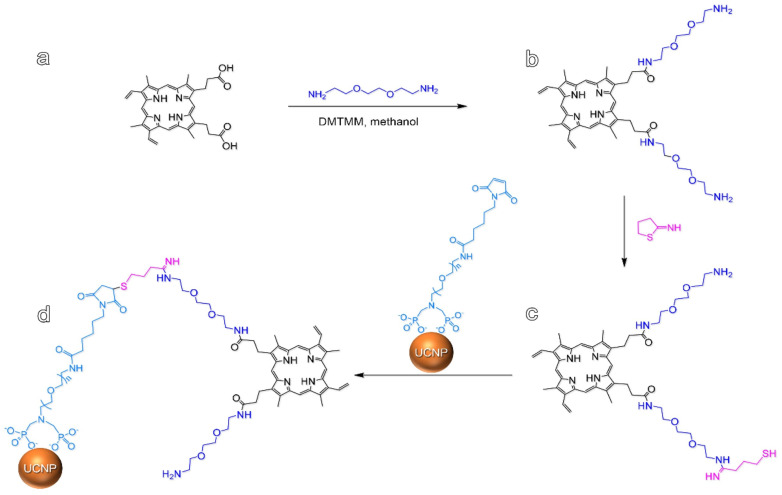
Synthesis routes of a probe based on upconversion nanoparticles (UCNPs). PpIX (**a**), porphyrin-jeffamine (PJ) (**b**), thiolated porphyrin-jeffamine (**c**), and the UCNPs–PJ probe (**d**) [96].

To increase the photoactivation efficiency of PpIX, Sun et al. chemically modified it with jeffamines and then covalently conjugated it to PEGylated upconversion NPs (UCNPs) (Figure 15) [96]. The UCNPs–PJ was more hydrophilic, able to generate ROS under the excitation of 980 nm NIR light, and displayed potential for targeting tumors. The UCNPs–PJ probe was evaluated in vitro using the human colon adenocarcinoma cell line (LS180), which is known to over-express LDL receptors that can capture porphyrin and facilitate UCNPs–PJ’s entrance into cells. Antiproliferative activity results showed that cell viability was close to zero at 1.5 mg/mL of UCNPs–PJ and a Live–Dead Cell Staining assay showed that most cancer cells were killed. Sun et al. [96] state that the light-triggered UCNP–PJ probes set the foundation for upconversion luminescence-mediated PDT anticancer nano-agents.

**Figure 16:**
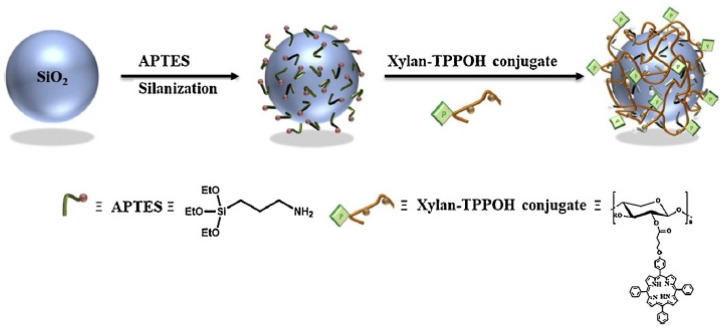
General procedure for the synthesis of PX SNPs. SIO_2_: silicon dioxide [97].

In our laboratory, Bouramtane et al. synthesized and characterized core-shell hybrid silica NPs (SNPs) based on xylan for targeted delivery of the PS 5-(4-hydroxyphenyl)-10,15,20-triphenylporphyrin (TPPOH) [97]. TPPOH was covalently linked with xylan, a natural polysaccharide, to increase the SNPs’ blood circulation half-life and stability, and then this xylan-TPPOH conjugate (PX) was attached to SNPs through ionic bonds to form PX SNPs (Figure 16). The in vitro cytotoxicity test of PX SNPs showed that they were 40-fold and 10-fold more effective against HCT116 cells and HT-29 cells, respectively, compared with free TPPOH. Moreover, TEM analyses of the morphology of HCT116 cells proved that these cells underwent apoptosis when treated with PX SNPs after irradiation only. In conclusion, PX SNPs have been successfully synthesized and showed high anticancer activity against CRC cell lines, which indicates that SNPs can be used as a drug carrier for PDT.

At the same time, in our laboratory, Bretin et al. used 80 nm silica NPs (SNPs) to enhance the selectivity and therapeutical potential of TPPOH [98]. They coated the SNPs with xylan. Results showed that vectorizing TPPOH with xylan-coated SNPs significantly ameliorated the efficacy of PS-PDT in vitro against CRC cells, as well as its in vivo tumor cytotoxicity. In vitro experiments on three human HT-29, HCT116 and SW620 CRC cell lines showed that TPPOH-X SNPs–PDT was 10.8-fold, 40.5-fold and 39.5-fold more cytotoxic than free TPPOH–PDT, respectively, and showed an enhanced cellular uptake and lysosome internalization with TPPOH-X SNPs compared to free TPPOH. Furthermore, Bretin et al. proved that by inhibiting PDT-related autophagy with 3-methyladenine (3-MA), TPPOH-X SNPs–PDT-induced cell apoptosis in the three cell lines was enhanced. The in vivo results in HT-29 xenograft models revealed that TPPOH-X SNPs+3-MA–PDT principally suppressed the tumor growth, and induced a higher level of cell apoptosis than TPPOH-X SNPs–PDT and free TPPOH–PDT, without inducing systemic toxicity. Taken together, Bretin et al.’s findings reveal that TPPOH-X SNPs seem to be a potent candidate for clinical tests.

Uppal et al. evaluated the photodynamic efficacy of chlorin *p6* bound to amine-modified silica nanoparticles (C*p6*-SiNP) in colon and oral cancer cell lines and found that there was an increase in the photodynamic activity of the C*p6*-SiNP complex compared to free chlorin *p6* (C*p6*) [75]. Fluorescence spectroscopy-based measurement showed similar intracellular uptake of free drug and C*p6*-SiNP, while measurements of phototoxicity showed that C*p6*-SiNP produced an approximately 85% higher phototoxic effect in colon carcinoma compared with free C*p6* upon exposure to red light. Fluorescence correlation spectroscopy experiments showed better binding of the drug with SiNP in serum media and thus an increase in photostability. To conclude, C*p6*-SiNP could provide better photodynamic efficacy compared to free C*p6*, but the toxic effect of SiNPs, when used at higher concentrations, is still a concern.

## 3. Gold Porphyrins for the Treatment of Colorectal Cancer

Among the metal-based compounds used for medical applications, porphyrin gold(III) complexes are receiving increasing attention as chemotherapies rather than PDT drugs because of their promising in vitro and in vivo anticancer activities [99,100,101,102]. It has been shown that porphyrin is one of the best-stabilizing ligands of gold(III) for avoiding its reduction, and porphyrin substitutions increase its complex solubility and cytotoxicity [103,104,105]. Activation of mitochondrial dysfunction and other apoptotic cellular events, and interaction with some specific enzymatic targets, such as selenol or thiol-containing thioredoxin reductase, proteasome, glutathione reductase, and peroxidase, have been proposed to account for the anticancer activities of gold complexes [106].

**Figure 17:**
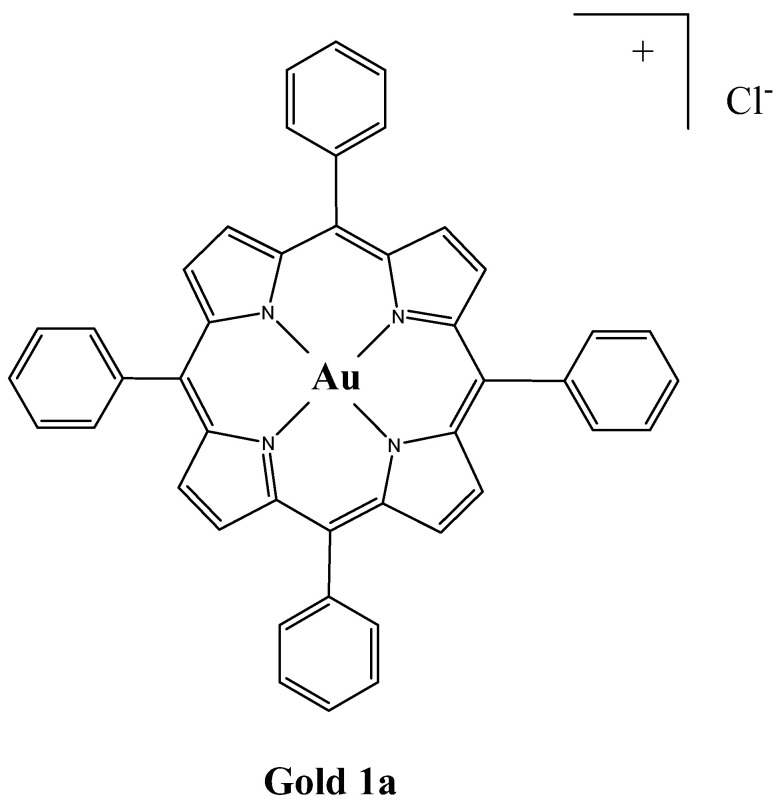
Chemical structure of compound Gold **1a** [111].

Interestingly, it was found that a compound named Gold **1a**, which is a gold(III) complex of *meso*-tetraphenylporphyrin ([Au(TPP)]Cl) (Figure 17), is 30-fold to 100-fold more cytotoxic than cisplatin in several human cancer cell lines, such as human cervix epitheloid cancer, nasopharyngeal carcinomas, and liver cancer [107,108,109,110]. These findings encouraged Tu et al. to explore the potential of Gold **1a** as an anticancer drug against CRC [102]. In vitro experiments on CRC cells (SW1116, Colo 205, CRL-238, CCL-2134, and HCT-15) showed that Gold **1a** caused a significant decrease in cell viability with IC_50_ ranging from 0.2 to 3.4 µM (8.7-fold to 20.8-fold lower than that of cisplatin), caused cell cycle arrest in the G0/G1 phase and induced apoptosis. The in vivo results on Colo 205 subcutaneous tumors proved that Gold **1a** inhibited tumor growth better than cisplatin with no obvious damage to the major organs of the mice [102]. These encouraging results make porphyrin Gold **1a** a promising chemotherapy drug.

**Figure 18:**
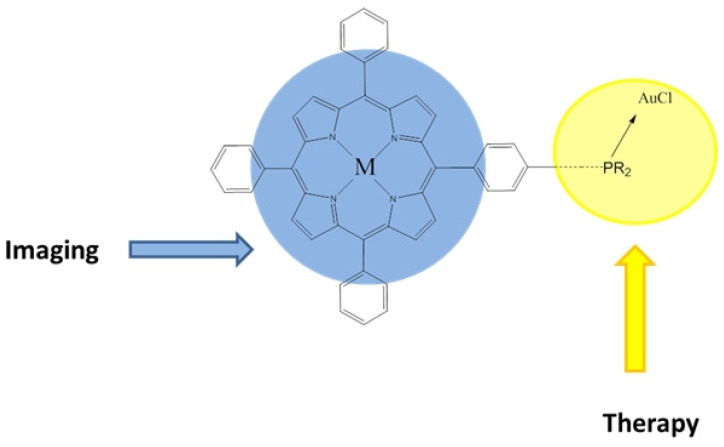
General structure of gold phosphine porphyrin derivatives as new theranostic agents (M = palladium or gold) [109].

Tasan et al. synthesized several phosphine porphyrin derivatives, such as free-base porphyrins, palladium(II) porphyrins, and two gold phosphine porphyrin (Figure 18) [112]. Antiproliferative activity tests were performed to determine the cytotoxicity of the different derivatives within two CRC cell lines (HCT116 and SW480). Only one water-soluble gold sulfonate complex showed significant toxicity with IC_50_ = 56 μM. Then, the different compounds were tested for photoactivation, and again only the gold sulfonate complex showed a significant decrease in IC_50_, to 14.8 μM. The significant difference in efficacy between the two gold phosphine porphyrin complexes was thought to be due to their aggregation behavior and cellular localization, since the detection of the two compounds via confocal microscopy after incubation with SW480 cells showed strong fluorescence, but the gold sulfonate complex showed very small aggregates which localized around cells, while the other complex formed aggregate-trapping cells [112]. Therefore, water-soluble gold phosphine porphyrin could be a potential metal-based theranostic whose activity could be increased by photoactivation.

**Figure 19:**
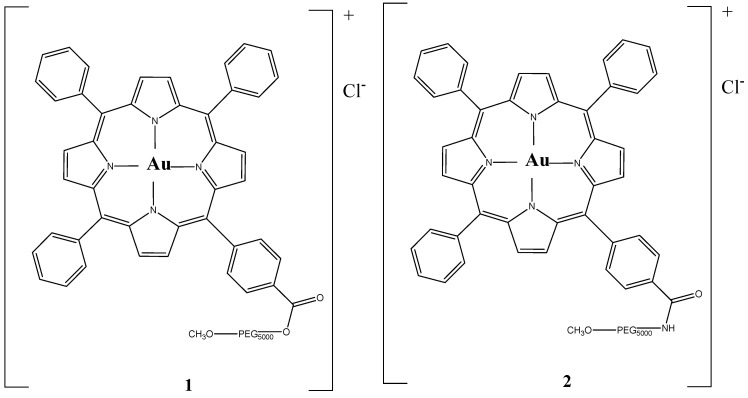
Chemical structures of [Au(TPP–COO–PEG5000–OCH_3_)]Cl (**1**) and [Au(TPP–CONH–PEG5000–OCH_3_)]Cl (**2**) [113].

In 2017, Chung et al. synthesized two anticancer gold(III) porphyrin–PEG conjugates, [Au(TPP–COO–PEG5000–OCH_3_)]Cl (**1**) and [Au(TPP–CONH–PEG5000–OCH_3_)]Cl (**2**) (Figure 19) [113]. The two drugs could self-assemble into nanostructures with sizes of 120–200 nm and the PEG made them more soluble, with prolonged blood circulation half-life and enhanced clearance mode [113]. The complex (**1**) demonstrated the best therapeutic results, such as good release property, fine tumor cell uptake and greater selectivity for inducing apoptosis in HCT-116 cells via inducing caspase-3/7 activation than in normal cells. The treated nude mice bearing HCT116 xenografts with 4 mg/kg of complex (**1**) showed a decrease in tumor weight (53%) and tumor volume (58%) with low systemic toxicity. In addition, Chung et al. proved that complex (**1**) was effective against the doxorubicin-resistant ovarian cancer cell line (A2780adr), which gives hope that it will also be effective against drug-resistant CRC cell lines [113]. Complex (**1**) showed promising drug characteristics and important anticancer effects that make it a good chemotherapy drug.

**Figure 20:**
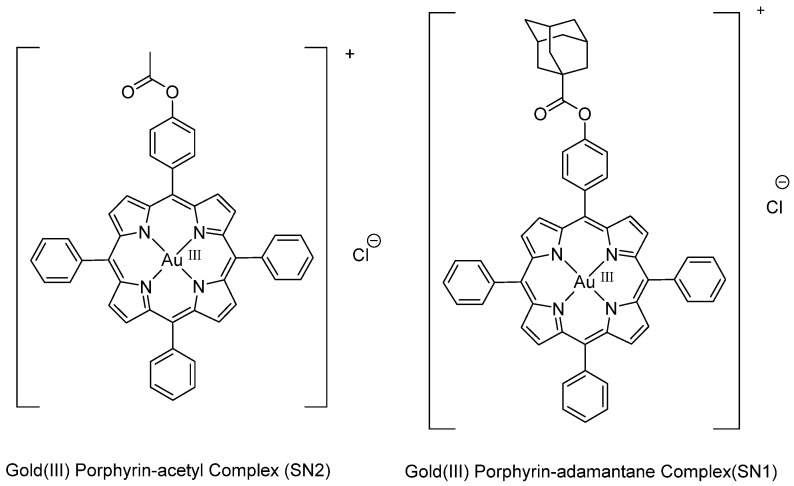
Chemical structure of gold(III) porphyrin-adamantane chloride complex (SN1) and gold(III) porphyrin-mono-acetate chloride complex (SN2) [114].

In previous work, we tested the anticancer activity of two novel gold(III) porphyrin complexes, gold(III) porphyrin-adamantane chloride (SN1) and gold(III) porphyrin mono-acetate chloride (SN2) (Figure 20), on the two CRC cell lines HT-29 and HCT-116 [114]. The two drugs showed significant antiproliferative effects on the CRC cell lines with IC_50_ ranging from 1.9 to 5.4 μg/mL at 48h of incubation, caused cell cycle arrest in the G2/M phase in HCT-116 cells, induced cell apoptosis (via activation of caspase cleavage and DNA fragmentation) and inhibited cell survival and proliferation pathways in both cell lines (deactivation of the PI3K/Akt pathway, dephosphorylation of ERK, and inhibition of NF-κB activation). Due to the importance of cyclooxygenase-2 (COX-2) in inducing chemoresistance in several cancers [115], we studied the effect of SN1 and SN2 on COX-2 expression and activity in HT-29 cells and found that SN1 and SN2 increased the expression and activity of COX-2. Therefore, we combined the drugs with NS-398 (a COX-2 specific inhibitor). HT-29 cells treated with the combined treatment showed increased DNA fragmentation [114]. Taken together, our findings revealed that SN1 and SN2 had very promising characteristics to be potent chemotherapeutic drugs against CRC.

**Figure 21:**
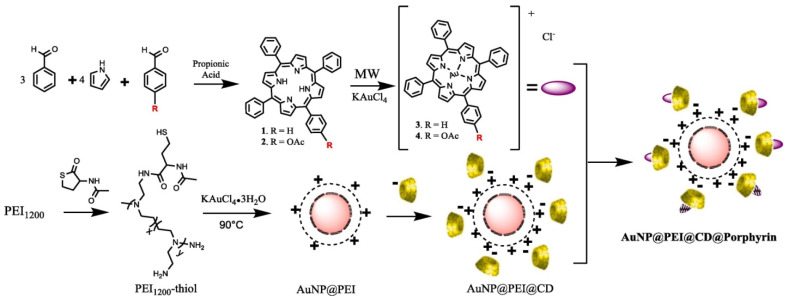
General pathway of AuNP@PEI@CD@Porphyrin synthesis [116].

To counteract the low solubility of anticancer gold(III) porphyrin drugs and increase their efficiency, Granet et al. in our research team constructed cyclodextrin scaffolds adsorbed on polyethyleneimine-coated gold NPs (AuNP@PEI@CD) (Figure 21) [116]. The hydrophobic tetrapyrrolic porphyrin compounds 5,10,15,20-tetraphenyl porphyrin and 5-(4-acetoxyphenyl)-10,15,20-triphenylporphyrin were encapsulated in the constructed scaffolds and their anticancer activity against the two CRC cell lines HT-29 and HCT-116 was compared to non-vectorized tetrapyrrolic macrocycles. The antiproliferative activity results showed that the IC_50_s of the two encapsulated gold porphyrins decreased significantly (3- and 5-fold in HT-29 cells and 1.5- and 2-fold in HCT-116 cells) compared with the corresponding non-vectorized tetrapyrrolic macrocycle. Granet et al. [116] successfully assembled a gold nanoparticle-based construct that amplified the therapeutical effects of gold(III) porphyrins and could be very useful in drug delivery.

**Figure 22:**
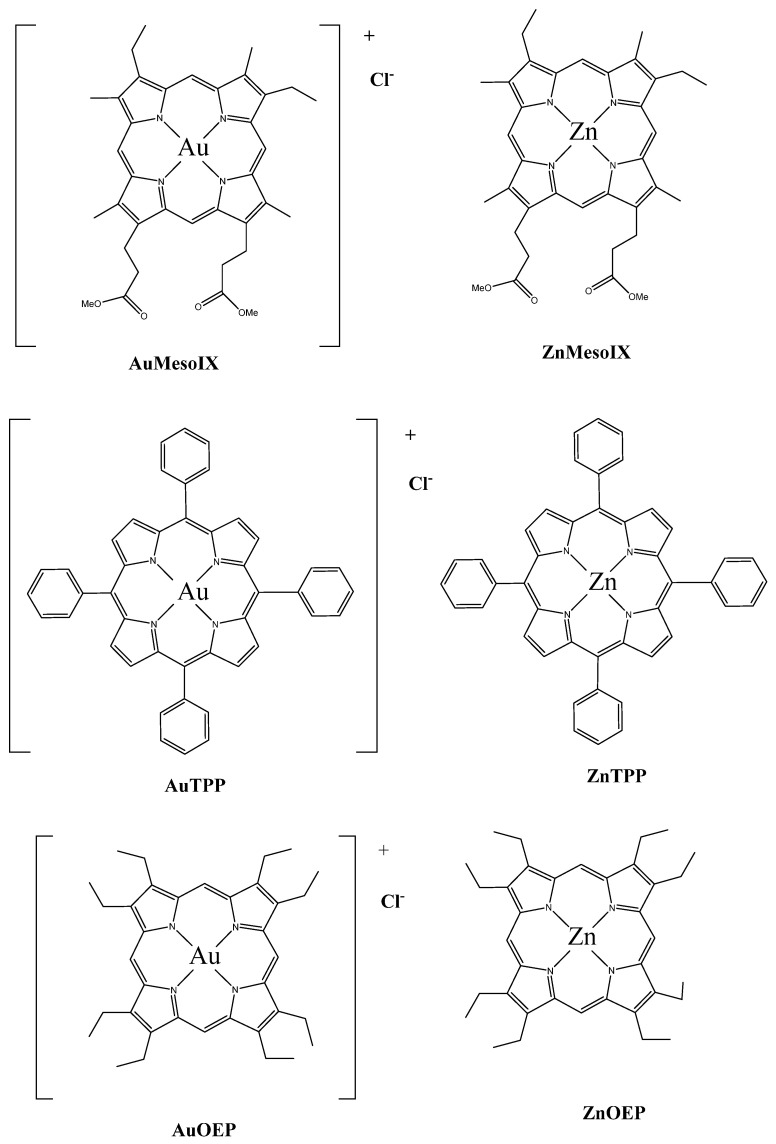
Chemical structures of gold(III) and zinc(II) porphyrin complexes [117].

In a recent study, Tong et al. examined and compared the anticancer effects of gold(III) meso-PpIX dimethyl ester (AuMesoIX) and its interactions with cellular thiols to two gold porphyrin complexes (octaethylporphyrin [AuOEP] and *meso*-tetraphenylporphyrin[AuTPP]) and three zinc(II) porphyrin complexes (Figure 22) [117]. The in vitro results illustrated that AuMesoIX had the best antitumor effects compared to the other compounds, since it was strongly cytotoxic against A2780cis ovarian cancer cells, HCT116 CRC cells, and NCI-H460 lung cancer cells, inhibited clonogenic growth, induced cell cycle arrest in the G0/G1 phase, and triggered apoptosis in the ovarian cancer cell line A2780 cells; it showed the best cellular uptake in the different cancer cell lines. Furthermore, it was found that AuMesoIX interacted with cancer-associated thiol-containing proteins such as peroxiredoxin III and protein deubiquitinases by conjugating to the cysteine residues and inhibiting their activities. The in vivo results showed that treatment of mice bearing HCT116 xenografts with AuMesoIX reduced tumor growth by 72% with no organ damage. On the whole, AuMesoIX, benefiting from its cysteine targeting ability, seems to be a potent anticancer drug.

## 4. List of Some Photophysical Properties of the Photosensitizers Described in this Review

With the goal of comparing the structures of some photosensitizers described in this review with regard to their efficiency on CRC treatment, the photophysical properties (ROS/singlet oxygen quantum) of various porphyrin/chlorin derivatives, the types of colorectal cancer cell lines tested, and the references are presented in Table 1.

## 5. Conclusions

CRC threatens the lives of many people worldwide. It is ranked the third most common cancer affecting both men and women worldwide and its five-year survival prognosis is highly dependent on the stage of the disease [9]. The survival percentage barely reaches 10% for patients with stage IV CRC. Unfortunately, common treatments come face to face with therapy resistance, which results in the low survival rates of CRC patients. For example, almost 50% of metastatic CRC patients are resistant to 5-FU-based chemotherapies [118], which are considered the standard treatment for advanced CRC [119]. Researchers continue to study ways to improve CRC therapies and progress in CRC treatment gives hope for finding less resistant drugs. Among the noninvasive medical techniques that show favorable results is PDT. To date, only Photofrin^®^ is FDA approved for use with PDT to treat or relieve the symptoms of esophageal cancer and non-small cell lung cancer. Many porphyrin-based drugs mentioned in this review are anticipated to be potential treatments based on their significant in vitro and in vivo therapeutic effects against CRC. However, clinical trials must first be conducted to confirm these results, benefiting from developments in nanotechnology and engineered nanomedicines, and researchers should begin developing targeted drug delivery platforms within pre-clinical and clinical trials and formulate standards in term of drug doses, drug administration methods, light doses, light types, efficacy, etc. for the sake of evolving CRC therapy.

## Figures and Tables

**Table 1:** Photosensitizers used in the treatment of colorectal cancer [48,52,76,91,92,95,96].

Photosensitizers Type	Abbreviation	Singlet Oxygen Quantum Yield	ROS	Cancer Cell Lines	Figure	Reference
Porphyrins	THPP	0.50	nc	WiDr, A375	1	[48]
	TBr_4_HPP	0.55	nc	WiDr, A375	1	[48]
	TI_4_HHP	0.58	nc	WiDr, A375	1	[48]
	TBr_12_HHP	0.50	nc	WiDr, A375	1	[48]
	2-TQP	0.62	nc	HT29	4	[52]
Chlorins	*m*-THPC	nc	***	HCT116, SW480	-	[76]
	Verteporphin (VP)	nc	***	HCT116, SW480	-	[76]
Porphyrins	TPAP	nc	***	HT29	10	[91]
	Zn PPIX mono adamantine **2Zn**	0.84	nc	HT29	11	[92]
	Zn PPIX diamantane **3Zn**	0.79	nc	HT29	11	[92]
	TAPP	nc	***	CT26	14	[95]
	PPIX-Jeffamine	nc	***	LS180	15	[96]
	UCNP PPIX-Jeffamine	nc	***	LS180	15	[96]

Nc: no data, ***: production of ROS.

## Data Availability

Not applicable.

## Sample Availability

Samples of the compounds are not available from the authors.
